# Supplementary material for: The COVID-19 Pandemic Increased Burnout and Bullying among Newly Graduated Nurses but Did Not Impact the Relationship between Burnout and Bullying and Self-Labelled Subjective Feeling of Being Bullied: A Cross-Sectional, Comparative Study
Source: Int J Environ Res Public Health. 2022 Feb 2;19(3):1730. doi: 10.3390/ijerph19031730 (PMC8835049; doi:10.3390/ijerph19031730)
Supplement: Supplementary file 1 [file ijerph-19-01730-s001.zip › ijerph-1585556-supplementary.pdf]

Suplementary file S1.

Table. Regression coefficients for the model of the moderating role of working as a newly graduated nurse before or during COVID-19 pandemic as a moderator between person-related bullying and disengagement

	B	*SE*	*t*	*p*	*95% CI*
Constant	11.15	2.01	5.56	<0.001	[7.20; 15.11]
Person-related bullying	3.38	0.87	3.89	0.001	[1.67; 5.09]
Working before or during COVID-19 pandemic	5.42	1.41	3.85	0.002	[2.64; 8.19]
Interaction	-1.84	0.60	-3.06	0.025	[-3.03; -0.65]

B – unstandardized regression coefficient; SE – standard deviation; *t* – *t-statistic;* CI – confidence interval

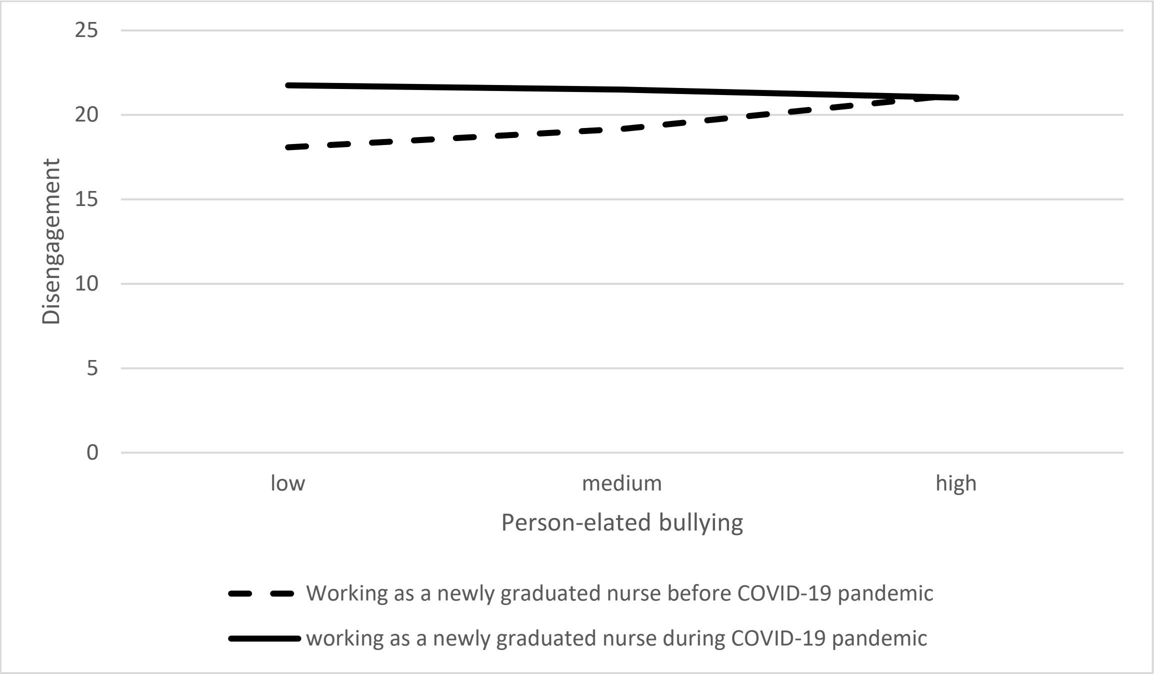

Figure. Relationship between person-related bullying and disengagement among newly graduated nurses who working before or during COVID-19 pandemic

Suplementary file S2.

Table. Regression coefficients for the model of the moderating role of working as a newly graduated nurse before or during COVID-19 pandemic as a moderator between work-related bullying and disengagement

	B	*SE*	*t*	*p value*	*95% CI*
Constant	10.47	2.33	4.48	<0.001	[5.86; 15.08]
Work-related bullying	3.39	0.93	3.63	0.003	[1.55; 5.23]
Working before or during COVID-19 pandemic	4.84	1.66	2.90	0.004	[1.55; 8.13]
Interaction	-1.46	0.66	-2.23	0.027	[-2.76; -0.17]

B – unstandardized regression coefficient; SE – standard deviation; *t* – *t-statistic;* CI – confidence interval

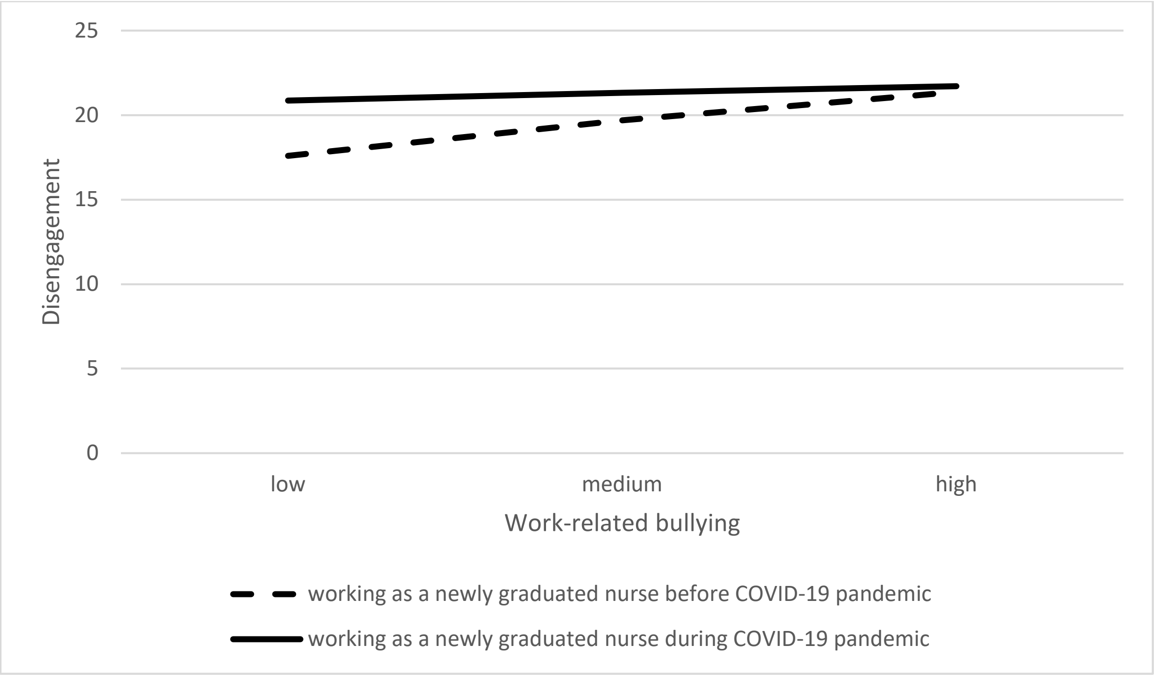

Figure. Relationship between work-related bullying and disengagement among newly graduated nurses who working before or during COVID-19 pandemic

Suplementary file S3.

Table. Regression coefficients for the model of the moderating role of working as a newly graduated nurse before or during COVID-19 pandemic as a moderator between intimidation-related bullying and disengagement

	B	*SE*	*t*	*p value*	*95% CI*
Constant	11.86	2.02	5.85	<0.001	[7.86; 15.85]
Intimidation-related bullying	4.50	1.30	3.44	0.007	[1.92; 7.08]
Working before or during COVID-19 pandemic	4.77	1.38	3.41	0.008	[1.98; 7.43]
Interaction	-2.24	0.89	-2.50	0.013	[-4.00; -0.47]

B – unstandardized regression coefficient; SE – standard deviation; *t* – *t-statistic;* CI – confidence interval

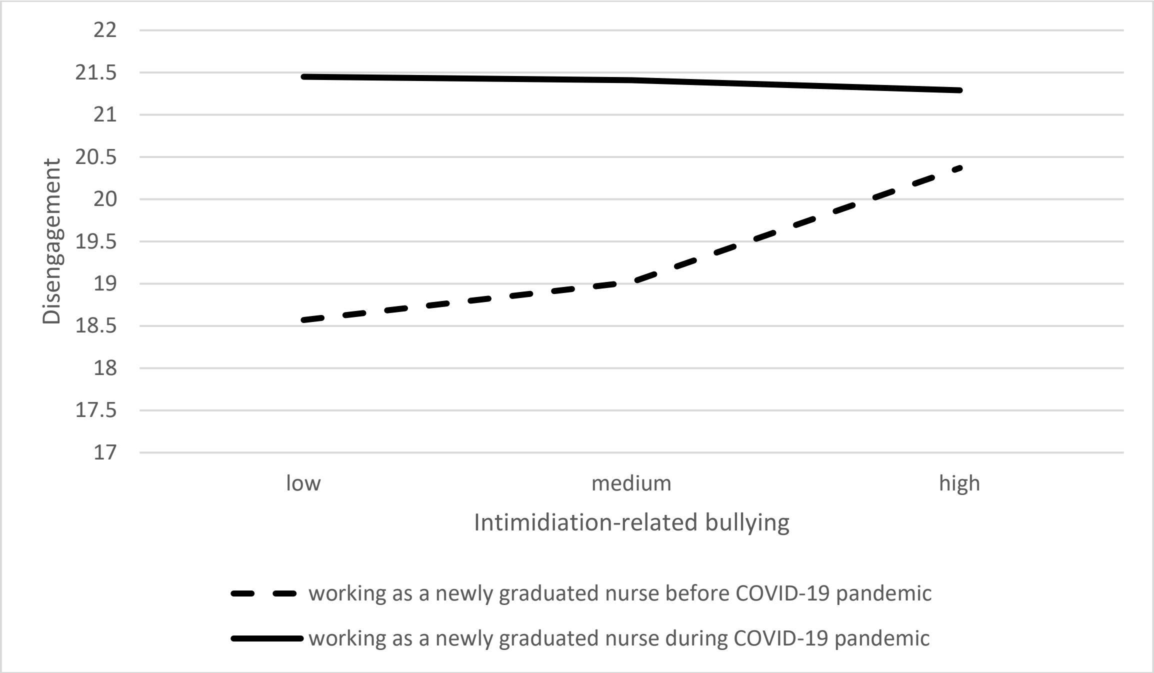

Figure. Relationship between intimidation-related bullying and disengagement among newly graduated nurses who working before or during COVID-19 pandemic

Suplementary file S4.

Table. Regression coefficients for the model of the moderating role of working as a newly graduated nurse before or during COVID-19 pandemic as a moderator between bullying and disengagement

	B	*SE*	*t*	*p value*	*95% CI*
Constant	9.77	2.26	4.31	<0.001	[5.30; 14.24]
Bullying	4.25	1.05	4.05	0.001	[2.19; 6.33]
Working before or during COVID-19 pandemic	5.91	1.62	3.65	0.003	[2.71; 9.10]
Interaction	-2.20	0.75	-2.94	0.036	[-3.67; -0.72]

B – unstandardized regression coefficient; *t* – *t-statistic;* CI – confidence interval

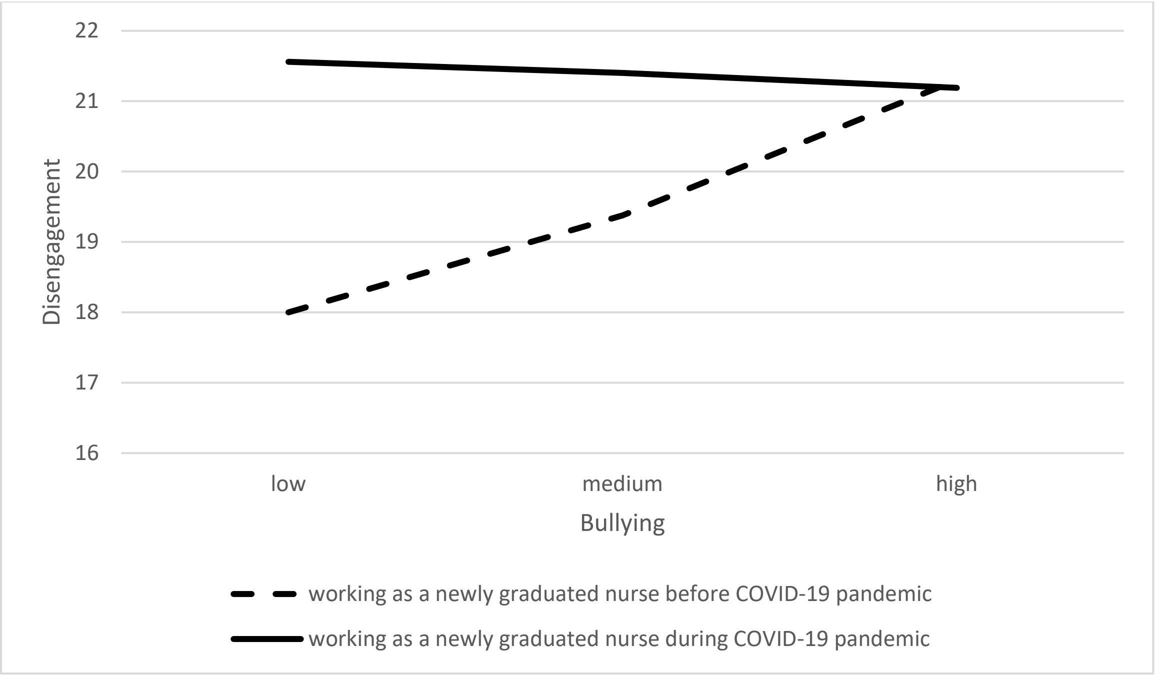

Figure. Relationship between bullying and disengagement among newly graduated nurses who working before or during COVID-19 pandemic
